# Graph theoretical approach to brain remodeling in multiple sclerosis

**DOI:** 10.1162/netn_a_00276

**Published:** 2023-01-01

**Authors:** AmirHussein Abdolalizadeh, Mohammad Amin Dabbagh Ohadi, Amir Sasan Bayani Ershadi, Mohammad Hadi Aarabi

**Affiliations:** Students’ Scientific Research Program, Tehran University of Medical Sciences, Tehran, Iran; Interdisciplinary Neuroscience Research Program, Tehran University of Medical Sciences, Tehran, Iran; Department of Neuroscience, Padova Neuroscience Center, University of Padova, Padova, Italy

**Keywords:** Multiple sclerosis, Remodeling, Diffusion MRI, Cognition, Graph theory

## Abstract

Multiple sclerosis (MS) is a neuroinflammatory disorder damaging structural connectivity. Natural remodeling processes of the nervous system can, to some extent, restore the damage caused. However, there is a lack of biomarkers to evaluate remodeling in MS. Our objective is to evaluate graph theory metrics (especially modularity) as a biomarker of remodeling and cognition in MS. We recruited 60 relapsing-remitting MS and 26 healthy controls. Structural and diffusion MRI, plus cognitive and disability evaluations, were done. We calculated modularity and global efficiency from the tractography-derived connectivity matrices. Association of graph metrics with T2 lesion load, cognition, and disability was evaluated using general linear models adjusting for age, gender, and disease duration wherever applicable. We showed that MS subjects had higher modularity and lower global efficiency compared with controls. In the MS group, modularity was inversely associated with cognitive performance but positively associated with T2 lesion load. Our results indicate that modularity increase is due to the disruption of intermodular connections in MS because of the lesions, with no improvement or preserving of cognitive functions.

## Author Summary

Network remodeling is an inherent property of the nervous system, diminishing the harm caused by lesions in multiple sclerosis (MS). Modularity has been proposed previously as a measure of network remodeling in several disorders. In this study, we studied modularity of structural networks in MS, and its associations with clinical and cognitive measures. We showed increased modularity in the patients that is associated with higher T2 lesion load and poor cognitive function; the latter became nonsignificant after covarying the lesion load. Our results indicate that modularity increase is due to the disruption of intermodular connections in MS because of the lesions, rather than a remodeling procedure resulting in a cognitive improvement.

## INTRODUCTION

Multiple sclerosis (MS) is a neuroinflammatory disease that causes demyelination in the central nervous system, seen as white and gray matte plaques (Frohman, Racke, & Raine, 2006). Although much has been done to comprehend its etiology, there are still some undisclosed areas requiring more attention. It seems that axonal loss in both forms of demyelination or degeneration disrupts information relay between gray matter areas, causing cognitive and functional impairments (Rocca et al., 2016).

Various studies indicate that the damaged brain tries to restore its normal functions by neuronal plasticity and axonal remodeling. Human MRI studies have demonstrated rewiring and activations in cortical segments of MS patients in both aspects of structural and functional forms, either because of intrinsic mechanisms or induced by rehabilitation (Prosperini & Di Filippo, 2019).

The expanding field of graph theoretical analysis helps neuroscientists better comprehend brain networks in health and disease. A graph consists of different nodes that are connected via pathways (i.e., edges). One of the methods to define a brain graph is to use cortical parcellations and subcortical regions as node definition and the wirings connecting these areas as the edges. Diffusion-weighted imaging (DWI) is a technique that can be used to identify the wirings (i.e., tracts) and their strength. Several metrics are derived from the resulting graph, which can be interpreted biologically (Rubinov & Sporns, 2010). Global efficiency is one of the integration measures that can determine the information flow efficiency of the whole brain. Along with that are other local segregation measurements, including modularity. Modules are a group of network nodes with stronger connections within the group and weaker connections between modules. This parameter can evaluate the optimization of the brain in varying environments (Girvan & Newman, 2002).

Various graph theory metrics have been applied in studies with MS patients. In a recent comprehensive review by Fleischer, Radetz, et al. (2019), both increase and decrease in global efficiency in structural and functional connectivity matrices of MS patients, and the presence or absence of association of such metrics with clinical status were reported. It seems that this heterogeneity in results arises from different analytic pipelines, confounding factors such as disease duration, and heterogeneity in the studied samples (including relapsing-remitting and primary or secondary progressive MS [RRMS, PPMS, and SPMS, respectively]). For example, in another study, lower global efficiency was reported in the MS group (including RRMS, PPMS, and SPMS) compared with healthy controls; however, there were no differences between RRMS and healthy controls (Charalambous et al., 2019). Interestingly, there is much less debate in the studies investigating modularity in MS. Increased modularity is a common finding in MS and is believed to represent the underlying network reorganization in response to lesions (Fleischer, Koirala, et al., 2019; Gonzalez-Escamilla et al., 2020); however, a few studies have investigated the clinical importance of it. In Fleischer et al. (2017), modularity was increased during the first year of RRMS diagnosis, without any change in clinical status, implying the probable protective role of network reorganization.

Our objective was to investigate the association between graph-derived metrics and cognitive/disability severity in RRMS patients, with a focus on the network remodeling assessment following lesions. To overcome the limitations of previous studies, we included a larger homogenous RRMS sample, to avoid the variability due to other phenotypes, and various clinical and cognitive measures. We hypothesize that modularity increase may underlie compensation of disease severity.

## METHODS

### Participants

We recruited our subjects from the Cross-Modal Research Initiative for Multiple Sclerosis and Optic Neuritis (CRIMSON) study. This study was conducted at MS Research Center, Sina Hospital, Tehran, Iran, which included RRMS and healthy controls (HC). More information regarding the inclusion and exclusion criteria and ethical approval of the study are reported elsewhere (Roostaei et al., 2019). In brief, the subjects had no prior history of head trauma and no psychiatric or neurologic disorders other than MS. All subjects had Expanded Disability Status Scale (EDSS) scores less than 6, and they had no history of relapse or corticosteroid therapy in the past three months. Before inclusion, all subjects signed informed consent, and this study was confirmed by the Ethical Review Board of Tehran University of Medical Sciences, Tehran, Iran.

### Evaluations

#### Cognitive scores.

All evaluations were done by expert researchers and neurologists in the CRIMSON study. Three cognitive tests were done: the Symbol Digit Modalities Test (SDMT; Brochet et al., 2008), California Verbal Learning Test (CVLT; Stegen et al., 2010), and Paced Auditory Serial Addition Test (PASAT; Rosti, Hämäläinen, Koivisto, & Hokkanen, 2007). These tests are widely used to evaluate cognitive function in MS patients. The procedure, evaluated cognitive functions, and evaluations included in the current study are explained in Table 1.

**Table 1.** Cognitive tests used in this study: The procedure, measures, and evaluated cognitive functions

**Test**	**Procedure**	**Evaluations**	**Cognitive function**
Symbol Digit Modalities Test (SDMT)	A symbol is assigned to each number 1 to 9 and is presented to the subject. Then, the subject is asked to pair a list of symbols to the numbers in 90 s.	Number of correct answers	Processing speed; attention
California Verbal Learning Test (CVLT)	A list of 16 nouns (list A) is read by the examiner to the subject and is recalled immediately, with a delay, cued or free, and with an interference (another list of 16 nouns; list B).	CVLT-TL (Total Learning): Total correct answers of five trials of the immediate recall	Declarative memory
		CVLT-LD (Long Delay): Number of words recalled from list A after 20 min	
Paced Auditory Serial Addition Test (PASAT)	A list of numbers is read by the computer, and the subject must add the last two numbers read.	Number of correct answers	Working memory; executive function

#### Clinical and disability scores.

We used the EDSS, MS Functional Composite (MSFC), and MS Severity Score (MSSS) as surrogates of disease severity and disability in our subjects. EDSS is scored by an expert MS clinician and is based on clinical examination and patient-reported dysfunctions in daily routines. It is widely used in clinical trials and routine patient evaluations (Kurtzke, 1983). MSFC is the mean of Z-score transformed scores of patients in three tests: timed 25-foot walk, 9-hole peg test, and PASAT (Fischer, Rudick, Cutter, & Reingold, 1999). Both MSFC and EDSS are cross-sectional evaluations that cannot address the speed of disability progression during disease duration. MSSS addresses this issue by correcting EDSS to disease duration based on an algorithm (Roxburgh et al., 2005).

### Imaging Acquisition

Using a Siemens Avanto 1.5T scanner, the following sequences were acquired: a magnetization prepared rapid-acquisition gradient-echo (MPRAGE) T1 sequence (repetition time [TR] = 2,730 ms, echo time [TE] = 2.81 ms, inversion time [TI] = 1,000 ms, field of view [FoV] = 256 mm, voxel size = 1 × 1 × 1 mm); a T2-weighted turbo spin echo with variable flip angle (TSE-VFL; TR = 3,200 ms, TE = 473 ms, FoV = 256 mm, voxel size = 1 × 1 × 1 mm); a fluid-attenuated inversion recovery (FLAIR) sequence (TR = 9,400 ms, TE = 83 ms, TI = 2,500 ms, FoV = 250 mm, voxel size = 1.3 × 1.0 × 3.0 mm); and a DWI sequence (TR = 9,500 ms, TE = 93 ms, voxel size = 2 × 2 × 2.1 mm, b-value = 1,000 s/mm^2^, in 64 diffusion directions) with three b0 volumes.

### Imaging Analysis

#### Lesion filling and lesion load.

We used lesion-filled T1 volumes and individual T2 hyperintense lesion load provided by the main investigators of the CRIMSON study. In brief, T2 hyperintense lesions were manually segmented, and individual lesion load was calculated. T2-to-T1 registration warp was then applied to the lesion mask to bring them into T1 space. Then, lesions were filled using the nearby normal-appearing white matter (Roostaei et al., 2019).

#### DWI preprocessing.

In order to preprocess and quality-check the diffusion data, the PreQual pipeline was built using the MRtrix3 (Tournier et al., 2019), FSL (Jenkinson, Beckmann, Behrens, Woolrich, & Smith, 2012), and advanced normalization tools (ANTs; Tustison et al., 2014) software packages. First, diffusion MR images were denoised using the MP-PCA function included with MRtrix3 (Cordero-Grande, Christiaens, Hutter, Price, & Hajnal, 2019; Veraart, Fieremans, & Novikov, 2016; Veraart, Novikov, et al., 2016). Gibbs ringing artifact reduction was attained by the local subvoxel-shifts method (Kellner, Dhital, Kiselev, & Reisert, 2016). Rician correction was performed with the moments method (Koay & Basser, 2006). The images were then intensity-normalized to the first image and concatenated for further processing. No reverse phase-encoded images were acquired, but corresponding lesion-filled T1 images of the subjects were available. Thus, a T1 image was used to generate a synthetic susceptibility-corrected b0 volume using SYNB0-DISCO, a deep learning framework by Schilling et al. (2019). This synthetic b0 image was used in conjunction with FSL’s topup to correct for susceptibility-induced artifacts in the diffusion data. FSL’s eddy algorithm was then used to correct for motion artifacts and eddy currents and to remove outlier slices (Andersson, Graham, Zsoldos, & Sotiropoulos, 2016; Andersson, Skare, & Ashburner, 2003; Andersson & Sotiropoulos, 2016; S. M. Smith et al., 2004). N4 bias field correction was then performed (Tustison et al., 2010). Lastly, the preprocessed data were fitted with a tensor model using the *dwi2tensor* function included with MRtrix3 using an iterative reweighted least squares estimator (Veraart, Sijbers, Sunaert, Leemans, & Jeurissen, 2013). This preprocessing pipeline’s quality was assessed qualitatively for gross errors and analyzed quantitatively using a three-step approach. In the first step, the preprocessed data were analyzed in accordance with the method outlined by Lauzon et al. (2013). The brain parenchyma without cerebrospinal fluid was masked in a restrictive manner by using an eroded brain mask generated on the average b0 image using the *bet2* function included with FSL (S. M. Smith, 2002). Then, the tensor fits of the masked data were backpropagated through the diffusion model to reconstruct the original diffusion signal. The goodness of fit for the tensor model was then assessed using a modified pixel chi-squared value per slice per volume. In the second step, the tensor fit was converted to a fractional anisotropy (FA) image (Basser, Mattiello, & LeBihan, 1994). The ICBM FA MNI atlas with 48 white matter tract labels provided with FSL was then non-rigidly registered to each subject’s FA image with the ANTs software package (Avants, Epstein, Grossman, & Gee, 2008; Hua et al., 2008; Mori, Wakana, Van Zijl, & Nagae-Poetscher, 2005; Wakana et al., 2007). The average FA of each tract was quantified and assessed for physiologic congruence. Lastly, the gradient orientations were visualized and checked using the *dwigradcheck* script included with MRtrix (Jeurissen, Leemans, & Sijbers, 2014).

#### DWI tractography.

The T1 image is corrected for intensity nonuniformity (Tustison et al., 2010) and skull-stripped, subcortical structures are segmented with FSL FIRST (Patenaude, Smith, Kennedy, & Jenkinson, 2011), and tissue types (gray matter, white matter, and cerebrospinal fluid) are labeled with FSL FAST (Zhang, Brady, & Smith, 2001). For anatomically constrained tractography, a nonlinear registration to MNI152 is computed (Tustison & Avants, 2013) and a five-tissue-type (5TT) image segmentation is derived. Cortical surface segmentations are derived from lesion-filled T1-weighted scans using FreeSurfer 6.0 (Fischl, 2012). The next step is to calculate an affine registration from native FreeSurfer space to T1 space. We used Desikan-Killiany parcellations provided by FreeSurfer (Desikan et al., 2006). Then, subcortical and cerebellar parcellations are nonlinearly registered to native DWI space (Tournier et al., 2019).

Structural connectomes are generated using MRtrix3. The iFOD2 algorithm and a three-tissue anatomically constrained tractography are used to create a tractography with 40 million streamlines (R. E. Smith, Tournier, Calamante, & Connelly, 2012; maximum/minimum tract length = 400/10 mm, FOD amplitude cutoff = 0.06, step size = 0.5). The resulting tractography’s second tract density image (TDI) is calculated for quality control. Then, full brain streamlines weighted by cross-sectional multipliers are rebuilt using spherical deconvolution guided filtering of tractograms (SIFT2; R. E. Smith, Tournier, Calamante, & Connelly, 2015). Then, a parcellation scheme is mapped to the reconstructed cross section weighted streamlines. Additionally, these are warped to DWI native space. Edge length matrices are also constructed, and the connection weights between nodes are defined as the weighted streamline count. All processing steps were done by the Micapipe pipeline (Cruces et al., 2022; https://micapipe.readthedocs.io/en/latest/).

### Connectivity Matrix

The connectivity matrix was obtained for each participant by dividing the streamline count by edge lengths, resulting in streamline density. Labels were based on Desikan-Killiany cortical parcellation plus subcortical structures and cerebellum, which generated 120 × 120 weighted connectivity matrices. We used Brain Connectivity Toolbox to calculate modularity (Louvain’s algorithm) and global efficiency for each individual (Rubinov & Sporns, 2010). More information regarding the graph metrics, their mathematical formula, and their possible interpretation in neurological studies can be found in numerous studies such as Rubinov and Sporns (2010). We used an iterative approach for modularity calculation. The iterations continued until the difference between the two calculations is less than the tolerance, which is chosen as 0.00001.

### Statistical Analysis

We used R statistical package v 4.0.4 (https://www.R-project.org/; R Core Team, 2019) for the statistical analysis of our data. First, subject demographics were analyzed using the Shapiro-Wilk statistical test to evaluate the normality of the data. Based on its results, a *t* test or Mann-Whitney U test was done to compare group (HC vs. MS) differences in demographics. We used analysis of covariance (ANCOVA) to compare group differences in graph metrics. Age and gender were inserted as covariates. To evaluate the time-dependent changes in graph metrics, we used Pearson’s or Spearman’s correlation tests based on the normal distribution of our data. Then, we confined our analysis to the MS group to analyze possible associations of graph metrics and markers of disease severity and cognitive tests. A general linear model was used with age and gender as covariates, with cognitive tests (SDMT, PASAT, CVLT-LD, CVLT-TL) or markers of disease severity (MSFC, EDSS, MSSS) included in the model as dependent variables and graph metrics (modularity and global efficiency) included as the predictor variable. To evaluate the association between T2 lesion load and graph metrics, we calculated normalized lesion load as total lesion volume divided by whole-brain volume for each individual. The normalized lesion load was then inserted into a general linear model with age and gender as covariates, and graph metrics as dependent variables. We used the Benjamini-Hochberg method to correct multiple-comparison correction in each category of tests (i.e., cognitive batteries and disease severity). Corrected *p* values less than 0.05 were considered significant.

## RESULTS

### Demographics

A total of 60 RRMS patients and 26 HC subjects were included in this study. Age did not follow a normal distribution (Shapiro-Wilk test *p* value for age in both groups < 0.001), so the Mann-Whitney U test was done. There were no significant differences of sex (*p* = 0.98) and age (*p* = 0.75) between the two groups. Subject demographics and values of cognitive and clinical evaluations are presented in Table 2.

**Table 2.** Between-group differences in demographics and cognitive and clinical evaluations. MSFC is calculated among 55 subjects who have their 9-hole peg test, 25-foot walk, and PASAT completed. HC = Healthy control. EDSS = Expanded Disability Status Scale. MSFC = Multiple Sclerosis Functional Composite. MSSS = Multiple Sclerosis Severity Score. SDMT = Symbol Digit Modalities Test. PASAT = Paced Auditory Serial Addition Test. CVLT = California Verbal Learning Test. CVLT-LD = CVLT–Long Delay. CVLT-TL = CVLT–Total Learning.

	HC *n* = 26	MS *n* = 60	*p* value
Demographics			
Gender (male (%))	4 (15.38)	11 (18.33)	0.98
Age (mean (*SD*))	30.65 (7.82)	30.75 (7.07)	0.75
Clinical evaluations (mean (*SD*))			
EDSS	–	2.76 (1.34)	
MSFC	–	0.00 (1.59)	
MSSS	–	4.37 (2.05)	
Cognitive evaluations (correct responses; mean (*SD*))			
SDMT	–	48.06 (15.94)	
PASAT	–	43.28 (10.98)	
CVLT-LD	–	11.53 (2.90)	
CVLT-TL	–	53.28 (8.21)	

### Between-Group Differences

Compared with HCs, RRMS subjects had higher modularity (mean (*SD*); for MS = 0.70 (0.01), for HC = 0.69 (0.01); *p* < 0.001) and lower global efficiency (mean (*SD*) × 1,000; for MS = 0.53 (0.03), for HC = 0.55 (0.04); *p* = 0.002).

### Graph Metrics and Disease Severity

**Figure 1.**
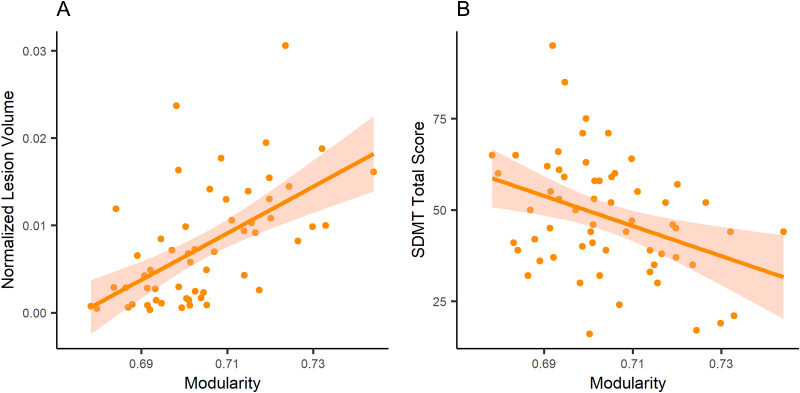
Linear models applied to an association of modularity with (A) normalized lesion volume and (B) correct answers in SDMT (Symbol Digit Modalities Test).

There were no significant associations between graph metrics and markers of clinical disease severity, except a negative association between modularity and normalized T2 lesion load. On the other hand, in the cognitive evaluations and after adjusting for multiple comparisons, modularity was significantly associated with SDMT (Table 3; Figure 1). Interestingly, there were no significant associations between global efficiency and clinical/cognitive evaluations. We also added normalized lesion volume as a covariate for associations of cognitive evaluations; there were no significant findings.

**Table 3.** Estimate (std. error) and *p* values of general linear models applied to identify significant associations between graph metrics (global efficiency and modularity), and clinical or cognitive evaluations of multiple sclerosis subjects, with age and gender as covariates. The *p* values are all corrected using Benjamini-Hochberg’s method. EDSS = Expanded Disability Status Scale. MSFC = Multiple Sclerosis Functional Composite. MSSS = Multiple Sclerosis Severity Score. SDMT = Symbol Digit Modalities Test. PASAT = Paced Auditory Serial Addition Test. CVLT = California Verbal Learning Test. CVLT-LD = CVLT–Long Delay. CVLT-TL = CVLT–Total Learning.

	Global efficiency		Modularity	
	Estimate (std. error)	*p* value	Estimate (std. error)	*p* value
Clinical evaluations				
EDSS	−3,017.185 (5,749.439)	0.80	21.756 (11.828)	0.29
MSFC	2,951.321 (2,551.787)	0.67	−3.395 (5.129)	0.80
MSSS	92.591 (8,796.878)	0.99	3.702 (18.592)	0.96
T2 lesion load	−19.153 (30.554)	0.80	0.262 (0.049)	<0.001***
Cognitive evaluations				
SDMT	61,450.748 (67,305.672)	0.41	−384.624 (133.871)	0.04*
PASAT	69,843.475 (49,542.373)	0.25	−167.882 (103.082)	0.25
CVLT-LD	3,364.148 (13,358.354)	0.80	−35.425 (26.507)	0.25
CVLT-TL	52,578.776 (35,848.396)	0.25	−109.18 (72.304)	0.25

* *p* < 0.05, *** *p* < 0.001.

Also, neither global efficiency nor modularity was significantly correlated with disease duration (Pearson’s *r* = 0.22 and −0.09; *p* = 0.08 and 0.48, respectively).

## DISCUSSION

We investigated modularity and global efficiency within patients with RRMS and HC subjects, showing higher modularity and lower global efficiency in patients with RRMS. We also demonstrated that higher modularity is associated with higher T2 lesion load and lower SDMT total scores. The association between modularity and SDMT disappeared after taking lesion load as a covariate in the statistical model. Our results indicate that modularity increase is due to the disruption of intermodular connections in MS because of the lesions, and have not resulted in preservation or improvement in cognitive functions.

Multiple sclerosis is considered a neuroinflammatory disease, and most of the current therapies target immune processes. While these treatments reduce disease progression, they cannot fully return the brain to the former undamaged state (Stampanoni Bassi et al., 2017). Recently, therapies such as remyelinating agents (Plemel, Liu, & Yong, 2017) have been proposed to try to regain function. These therapies can also be influenced by factors that affect neuroplasticity, such as immune responses (Golia et al., 2019) and even hormones (such as thyroid hormones; Remaud, Gothié, Morvan-Dubois, & Demeneix, 2014). Thus, developing novel biomarkers for plasticity can be of great importance in evaluating regain-of-function therapies. Modularity is proposed as a promising tool and has shown promising results in brain injury (Han, Chapman, & Krawczyk, 2020).

Network changes in RRMS have been reported in previous studies conducted using both structural and functional connectivity measures (Fleischer, Radetz, et al., 2019). Decreased global efficiency using connectivity matrices derived from fMRI is a common finding in RRMS and is also associated with disease severity (Rocca et al., 2016; Tommasin et al., 2020). Usually, in fMRI studies, the Pearson correlation coefficient of time series between each pair of regions is used as a measure of connectivity between two regions. However, studies based on DWI-derived connectivity matrices have shown different results. While some studies reported a decrease in global efficiency in RRMS (Charalambous et al., 2019; Shu et al., 2011), others have shown no differences between HC and RRMS (Fleischer et al., 2017; Muthuraman et al., 2016). Our results support the former studies. Interestingly, both global efficiency and modularity were not correlated with disease duration in our RRMS group, probably showing no time-dependent changes in these metrics in RRMS.

According to the hierarchical Newman’s equation of modularity or the optimization-based Louvain’s algorithm, increased modularity can be achieved either by increasing intramodular connections (i.e., reorganization) or through loss of intermodular connections (i.e., damage caused by lesions; Blondel, Guillaume, Lambiotte, & Lefebvre, 2008; Newman & Girvan, 2004). In other words, in the former, the brain generates new connections and modules to regain its function. In the latter, lesions damaging local and global connections result in more collection of locally connected nodes (i.e., modules). Modularity increase due to remodeling has been shown in traumatic brain injury (Han et al., 2020), as a developmental process (Chen & Deem, 2015), and as a marker of improvement in the narrative production of aphasic patients (Duncan & Small, 2016). However, in another study, higher modularity in the left hemisphere in post-stroke patients was associated with poor clinical outcomes (Marebwa et al., 2017), probably reflecting the lesions damaging the intermodular (rather than intramodular) connections, causing increased modularity. Increased modularity in MS patients compared with HC has been reported previously. Fleischer et al. (2017) showed that RRMS subjects had higher modularity than HC, at baseline, and in the follow-up. This increase in modularity was not associated with disease severity using EDSS. Modularity based on gray matter covariance matrices was also associated with T2 lesion load and disease duration; however, no results were reported for such correlation using DWI-based networks (Fleischer et al., 2017). In another study, modularity was lower in clinically isolated syndrome (CIS), but higher in RRMS patients compared with HC (Kocevar et al., 2016). In a similar study, Muthuraman et al. (2016) showed increased modularity in RRMS compared with CIS, and both compared with HC. A recent study using higher order DWI models also revealed this increase in modularity. They also reported a negative association between modularity and SDMT (Tur et al., 2019). In a relevant study using fMRI, higher modularity was evident in early MS patients, but similar to our findings, it was inversely correlated with a cognitive function (Gamboa et al., 2014). We report this negative association between modularity and cognitive function of processing speed and attention based on SDMT. Of great importance, this association became nonsignificant after adjusting for T2 lesion load. This indicates that reduced SDMT performance can be the result of widespread lesions across the brain. Based on this finding and also the negative association of modularity with T2 lesion load in our sample, we postulate that increased modularity based on tractography-based networks reflects the preferable disruption of intermodular connections in RRMS, which leaves an isolated collection of nodes (i.e., generating modules) or a failed remodeling, rather than a successful remodeling procedure.

Despite implementing novel graph theoretical approaches to investigate remodeling in MS, our study is faced with limitations. Our study has a cross-sectional research design that lacks the power of longitudinal studies to better address the main question of the current study. Tractography in MS has always been problematic because of the random presence of lesions, causing a sudden stop in tractography or even spurious tracts. Any algorithm applied has its caveats and benefits, and currently, there is no gold standard algorithm developed for such situations (Lipp et al., 2020). To overcome this issue, we used the PreQual pipeline, which uses several applications to improve volumetric segmentation, cortical parcellation, tractography, and connectivity matrix generation. It also has several quality-control checkpoints to ensure the minimizing of analytic pitfalls. Adding another imaging method, such as fMRI, and comparing the graph metrics derived from the fMRI-based with DWI-based networks may add some more perspective to our findings. Also, since we did not have cognitive evaluations done on the HC, we do not know for sure whether the observed association between cognitive measures and modularity is limited to MS or whether it is also applicable to HC. Future studies adding cognitive measures in HC and applying group × cognition interaction analysis may address this problem.

In conclusion, our study showed that increased modularity is the result of disrupted intermodular connection due to lesions and has not resulted in regaining cognitive function. Future longitudinal studies using both structural and functional imaging are suggested.

## ACKNOWLEDGMENTS

We gratefully thank Dr. Parvin Pasalar, the former director of the Students’ Scientific Research Center of Tehran University of Medical Sciences, Tehran, Iran, for her role in supporting and mentoring authors (Namely A. H. A. and A. O.).

## AUTHOR CONTRIBUTIONS

AmirHussein Abdolalizadeh: Conceptualization; Formal analysis; Investigation; Methodology; Project administration; Writing – original draft; Writing – review & editing. Mohammad Amin Dabbagh Ohadi: Formal analysis; Writing – original draft. Amir Sasan Bayani Ershadi: Writing – original draft. Mohammad Hadi Aarabi: Formal analysis; Methodology; Writing – original draft.

## FUNDING INFORMATION

Mohammad Hadi Aarabi, euSNN European School of Network Neuroscience, Award ID: MSCA-ITN-ETN H2020-860563.

## TECHNICAL TERMS

Multiple sclerosis (MS): An autoimmune disorder of the central nervous system causing motor, sensory, and cognitive symptoms.
Demyelination: Destruction of the myelin sheaths that surround the axons or dendrites, resulting in impaired neural impulse conduction.
Relapsing-remitting multiple sclerosis (RRMS): The most common form of MS; it is marked by periods of remission divided by attacks (i.e., relapse) of the disease.
Primary progressive multiple sclerosis (PPMS): A form of MS in which the patient’s status deteriorates from the first attack, causing severe disability within a few years.
Secondary progressive multiple sclerosis (SPMS): RRMS patients fail to remit to a nearly healthy status after several years of attacks, showing severe disability.
Corticosteroid: A drug group that reduces the tissue damage caused by the immune system. They are used in multiple sclerosis attacks.
